# Editorial: Emerging Biomarkers for NSCLC: Recent Advances in Diagnosis and Therapy

**DOI:** 10.3389/fonc.2021.694578

**Published:** 2021-05-14

**Authors:** Umberto Malapelle, Etienne Giroux Leprieur, Paul Takam Kamga, Marius Tresor Chiasseu, Christian Rolfo

**Affiliations:** ^1^ Department of Public Health, University of Naples Federico II, Naples, Italy; ^2^ Department of Respiratory Diseases and Thoracic Oncology, APHP-Hopital Ambroise Pare, Boulogne-Billancourt, France; ^3^ EA 4340 BECCOH, UVSQ, Université Paris-Saclay, Boulogne-Billancourt, France; ^4^ Cellular Neuroscience, Neurodegeneration and Repair Program, Yale University School of Medicine, New Haven, CT, United States; ^5^ Departments of Neurology, Yale University School of Medicine, New Haven, CT, United States; ^6^ Thoracic Medical Oncology, Marlene and Stewart Greenebaum Cancer Center, University of Maryland, Baltimore, Baltimore, MD, United States

**Keywords:** biomarkers, NSCLC, diagnosis, therapy, NGS

The advent of precision medicine and predictive molecular pathology has significantly modified the clinical management of patients with non-small cell lung cancer (NSCLC). A plethora of different biomarkers has been approved for predictive molecular purposes (Zhu et al.; Li et al.; Lin et al.). In this scenario, molecular techniques able to optimize the limited amount of nucleic acids extracted from small tissue and/or liquid biopsy samples are essential for the different clinically relevant biomarkers evaluation. Next generation sequencing (NGS) is a fascinating molecular approach able to analyze different gene alterations from different patients, simultaneously, starting from low input material. However, it should be remembered that a careful process of validation and harmonization of wet and dry procedures are strongly warranted (Malapelle et al.). Beyond the administration of tyrosine kinase inhibitors, a high percentage of NSCLC patients without any driver alteration can benefit from the administration of immune-checkpoint inhibitors (ICIs). Despite the established role of the evaluation of programmed death-ligand 1 (PD-L1) expression through immunohistochemistry or immunocytochemistry on tissue specimens, several other biomarkers are currently under investigation. Among these, tumor mutational burden (TMB) evaluated on tissue samples is the most commonly studied. However, TMB evaluation suffers from some technical issues. Thus, the adoption of surrogate biomarkers, such as *MSH2* expression (Jia et al.), may be a valid option. In addition, blood TMB evaluation may be a valid opportunity to assess TMB status and monitor ICIs response (Friedlaender et al.). Liquid biopsy adoption is increasing due to a not negligible percentage (about 30%) of NSCLC patients who do not have tissue availability for molecular analysis. Beyond the predictive purposes, liquid biopsy may play a pivotal role in the early diagnosis and prognosis evaluation of lung cancer (Dong et al.; Xi et al.). In the setting of prognostic biomarkers, many data have emerged on NSCLC. In particular, micro RNA (miRNA) 1323 with high expression in lung adenocarcinomas, promoting cancer cell migration, is associated with a poor prognosis (Zhao H et al.). Other prognostic biomarkers are currently under investigation, in particular those related to metabolic reprogramming, extracellular matrix, and tumor microenvironment remodeling (Bi et al.; Yang et al.; Czarnecka et al.; Ma et al.; Ahmed). Another interesting field of investigation concerns the development of prognostic models (Wu L-L et al.) and immunoscoring strategies to stratify early stage patients (Zhao Z et al.). Finally, careful attention should be paid to the novel approaches related to machine learning algorithms used to predict lymph node involvement in early T stage patients (Wu Y et al.), the possibility to isolate and characterize stem-like cells (Masciale et al.), and the possibility to adopt a radiomics-based nomogram to predict *EGFR* mutation subtypes (Zhao W et al.).

Taken together, the papers published in Research Topic “Emerging Biomarkers for NSCLC: Recent Advances in Diagnosis and Therapy” represent a critical discussion focused on the role of different novel biomarkers for both predictive and prognostic purposes.

## Author Contributions

Writing the original draft: UM and CR. All authors contributed to the article and approved the submitted version.

## Conflict of Interest

UM has received personal fees (as consultant and/or speaker bureau) from Boehringer Ingelheim, Roche, MSD, Amgen, Thermo Fisher Scientifics, Eli Lilly, Diaceutics, GSK, Merck and AstraZeneca, unrelated to the current work. CR reported receiving research grants at Antwerp University Hospital, Belgium, from Novartis and Sanofi, receiving speaker fees from Guardant Health, Merck Sharp & Dohme, and Novartis, receiving scientific advisor fees from Mylan, serving on a steering scientific committee for Oncompass, and participating in research collaborations for OncoDNA and Biomark Inc, unrelated to the current work. EG has received personal fees (as consultant and/or speaker bureau) from AstraZeneca, Boehringer Ingelheim, Bristol-Myers-Squibb, Eli Lilly, MSD, Novartis, Roche, Takeda, and research grants (institution) from AstraZeneca, Bristol-Myers-Squibb, and Roche.

The remaining authors declare that the research was conducted in the absence of any commercial or financial relationships that could be construed as a potential conflict of interest.

